# A Two-Step Strategy for Fabrication of Biocompatible 3D Magnetically Responsive Photonic Crystals

**DOI:** 10.3389/fchem.2019.00026

**Published:** 2019-02-01

**Authors:** Hui Liu, Caiqin Wang, Peixi Wang, Nan Liu, Qingfeng Du

**Affiliations:** ^1^School of Public Health, Lanzhou University, Lanzhou, China; ^2^General Practice Center, Nanhai Hospital, Southern Medical University, Foshan, China

**Keywords:** 3D, magnetically responsive photonic crystals, biocompatible, superparamagnetic nanosphere, self-assembling

## Abstract

Extremely stable and biocompatible 3D magnetically responsive photonic crystals (MRPCs) are successfully prepared in aqueous solution. Classic hydrothermal synthesis was applied for preparation of the Fe_3_O_4_@C core. Modified Stöber method was then employed for synthesis of the different size of Fe_3_O_4_@C@SiO_2_. Unlike the traditional magnetic nanoparticles, the highly negative charged superparamagnetic nanospheres (SMNs), i.e., the double-shell structure Fe_3_O_4_@C@SiO_2_ are capable of rapidly self-assembling into 3D MRPCs with full visible and various colors that can be periodically and reversibly tuned under different kinds of external magnetic fields (EMFs) within 1 s. The assembling behavior and mechanism of the 3D MRPCs under EMF were monitored and analyzed. The preparation is simple and the size of the SMN is easily controllable by adjusting the amount of catalyst. Compared with the previous works, the synthesized 3D MRPCs are hydrophilic, and exhibit extremely high stability after 6-month storage. To conclude, our study provides an effective two-step strategy for fabrication of biocompatible 3D MRPCs and it reveals great potentials in biological fields.

## Introduction

Photonic crystals (PCs) are considered as one of the tunable optical nanomaterials and exhibit high brightness and saturation, permanent color, and an iridescent effect (color changes with viewing angles) (Vukusic and Sambles, 2003; Teyssier et al., 2015). Owing to the existence of a photonic band gap, they are capable of confining, controlling and prohibiting the propagation of light by a band of frequency. The traditional self-assembling methods of PCs have been employed, such as centrifugal force (Ma et al., 2013), electrostatic interaction (Ge et al., 2007b; Walker et al., 2010), electrophoretic deposition (Rogach et al., 2000) and capillary force (Jiang et al., 1999; Bowden et al., 2001; Shiu et al., 2004), electric field (Forster et al., 2011), and microfluidic method (Yu et al., 2012), etc. However, many challenges of the previously reported PCs are still existing, such as time-consuming, slow response, narrow band gap adjustment, uncertain orientation or size and incomplete reversibility, etc.

Recently, rapid and efficient preparation of high qualified PC has received much attention in material sciences (Askar et al., 2016). However, most PCs are poorly biocompatible and organic solvents are frequently used in the synthesis processes, which is not suitable for biological applications. Thus, the design of building blocks with novel morphology and development of unique periodic structures for tuning the biocompatibility of PCs cannot to be ignored. Magnetic field has been regarded as an instantaneous and effective stimulus to induce the self-assembling of superparamagnetic nanospheres (SMNs) into periodic colloidal orderly arrays and tune the diffraction of photonic structures in a contactless manner, benefiting from the nature of magnetic interactions (He et al., 2012b; Wang et al., 2013). which is superior to traditional self-assembly method. SMNs have been widely used in many fields, especially the biological fields (Chen et al., 2015; Shen et al., 2015; Ulbrich et al., 2016; Lu et al., 2017). Under magnetization inducing under the external magnetic field (EMF), the SMNs with appropriate sizes can be self-assembled into magnetically responsive PCs (MRPCs) by optical confining band gap. Even though MRPCs have been explored in the past years, most of them couldn't assembled in aqueous solution (Wang et al., 2010b, 2011a,b; Luo et al., 2014, 2017) which limited the applications in the biological field. Therefore, it is necessary to prepare novel hydrophilic and stable MRPCs with the advantages of high production efficiency and high-quality with rapid, time-saving, and convenient preparation for promising application in biological fields. This novel biocompatible and self-assembly technology can expand the application range of PCs, and the biocompatibility of magnetic induction of high-qualified MRPCs as a kind of new intelligent materials in security, physical and biological sensors, chemical pollutants detection and monitoring is vital of scientific and application value.

The electric dipole-dipole interactions between SMN particles and the concentration of particles play important roles during the assembly process of MRPCs under EMF, from one dimensional (1D) chain-like structures as the simplest ordered state to two dimensional (2D) sheets and three dimensional (3D) quasi-close-packed structures (Wang et al., 2013). The highly charged surfaces provide sufficient long-range interparticle electrostatic repulsion, which balances the magnetic dipole-dipole attraction and establishes a force equilibrium within the chain-like structures (Wang and Yin, 2016) In this work, we have prepared extremely stable and highly charged Fe_3_O_4_@C@SiO_2_ SMNs, i.e., the 3D MRPCs which are capable of orderly self-assembling in water and biocompatiblity under EMF. Due to the inertness of the middle carbon layer, they have the superior property of high stability and can be stored in water for at least 6 months which gains an advantage over the other MRPCs prepared by our previous work (Tang et al., 2018). The assembling behavior and mechanism of the 3D MRPCs under EMF were monitored and analyzed. The critical point for the preparation of 3D MRPCs lies in the establishment of a balance among the dipole-dipole attractive forces, exclusion forces, and dipole-dipole electrostatic repulsive forces. This approach is able to endow the SMNs with a long-term stability almost independent of ionic strength, pH-value and solvent polarity. Compared with the previous works (Whitesides and Boncheva, 2002), our synthesized SMNs are hydrophilic, and exhibit extremely high stability after 6-month storage.

## Materials and Methods

Ferrocene (>98%) was obtained from Sigma-Aldrich (St. Louis, MO, USA). Tetraethyl orthosilicate (TEOS) was obtained from J&K Scientific Ltd (Beijing, China). All the other chemicals were of analytical-reagent grade. All the water used throughout the experiment was purified using the Milli-Q system (Millipore, Bedford, MA, USA), which had a minimum resistivity of 18 M·Ω cm.

### Fabrication of the 3D MRPCs by the Two-Step Method

Fe_3_O_4_@C@SiO_2_ were synthesized via a two-step method. In the first step, we used classic hydrothermal synthesis the core of Fe_3_O_4_@C (Wang et al., 2010a) with some modifications. 0.7 g of ferrocene was dissolved in 70 mL acetone, sealed and sonication for 10 min; and 3 mL hydrogen peroxide was added into the mixture, sealed and magnetically stirred for 30 min. Then it was transferred to the polytetrafluoroethylene-lined autoclave and heated at 210°C for 48 h. After that, the mixture was naturally cooled to room temperature (RT) and repeatedly sonication washed with ethanol. Finally, the products (Fe_3_O_4_@C) were magnetically attracted and dried at 60°C. In the second step, a modified typical Stöber method was employed for the synthesis of a different size of Fe_3_O_4_@C@SiO_2_. 0.1g of Fe_3_O_4_@C was dispersed in the mixed solution of 50 mL ethanol and 10 mL water in a three-neck flask; and after a vigorous sonication for 10 min and 500 rpm stirring for 10 min at 25°C, 0.6 mL (1 mL or 1.5 mL) of ammonia solution (the catalyst) was added to the above solution and stirred for 10 min, then 0.6 mL tetraethyl orthosilicate was added into the mixed solution, and maintained 500 rpm stirring for 12 h at 25°C. After that, an 8,000 rpm centrifuge was carried out for 10 min and the precipitate was collected, washed several times by water and dispersed in 5 mL water.

### Characterizations

The core-shell characteristic of SMNs microstructures were observed by transmission electron microscope (TEM) (Hitachi, H7650, Japan) at a voltage of 100 kV and scanning electron microscope(SEM) (ZEISS, Supra 55-VP, Germany) in high vacuum mode at accelerating voltage of 10 kV. Zetasizer (Zeta 3000HS, Malvern, UK) was used to measure the surface charge (ξ) and size distribution(d) of the particles at 25°C. The reflectance spectra of the MRPC was recorded by optical fiber spectrometer (HR2000, Ocean Optics, USA). X-ray diffraction (XRD) (D/MAX-2500 diffractometer, Rigaku, Japan, 18 kV) with a Cu Kα irradiation (λ = 1.5405 Å) and X-ray photoelectron spectroscopy (XPS) (Kratos Analytical Ltd, Axis Ultra DLD, UK) were combined to measure the crystal structure and elementary composition of the SMNs. The scanning range was 10°–70° and the scanning interval was 0.02°/2θ. Fourier transform infrared spectrum (FT-IR) (Nicolet NEXUS 870, Thermo-Fisher, USA) was used to determine the groups composition of the SMNs. The wavelength range is 450–4,000 cm^−1^ with a resolution of 4 cm^−1^. The corresponding magnetic hysteresis loops of the SMNs were obtained by physical property measurement system (PPMS®-9, Quantum, USA). Dark-field optical microscopy (Eclipse Ci-S/Ci-L, Nikon, Japan) was employed to record the assembling process of the SMNs with/without magnet.

## Results and Discussion

As we all know that the nanocrystal-Fe_3_O_4_ can be attracted by EMF and is easily to be aggregated. In the state of aggregation, MRPCs cannot be formed. After coating of C layer by the hydrothermal method, the aggregation between Fe_3_O_4_ particles could be avoided. It is very important to provide proper surface coating and develop an effective protection strategy to keep the stability of magnetic iron oxide SMN. The silane agent is often considered as a precursor for the direct modifying on the surface of iron oxide MNS to generate high density of surface functional groups (Chen et al., 2016) for keeping the biocompatibility as well as binding the various biological materials by silica (SiO_2_)-coating modification (Ashtari et al., 2005).

**Figure 1:**
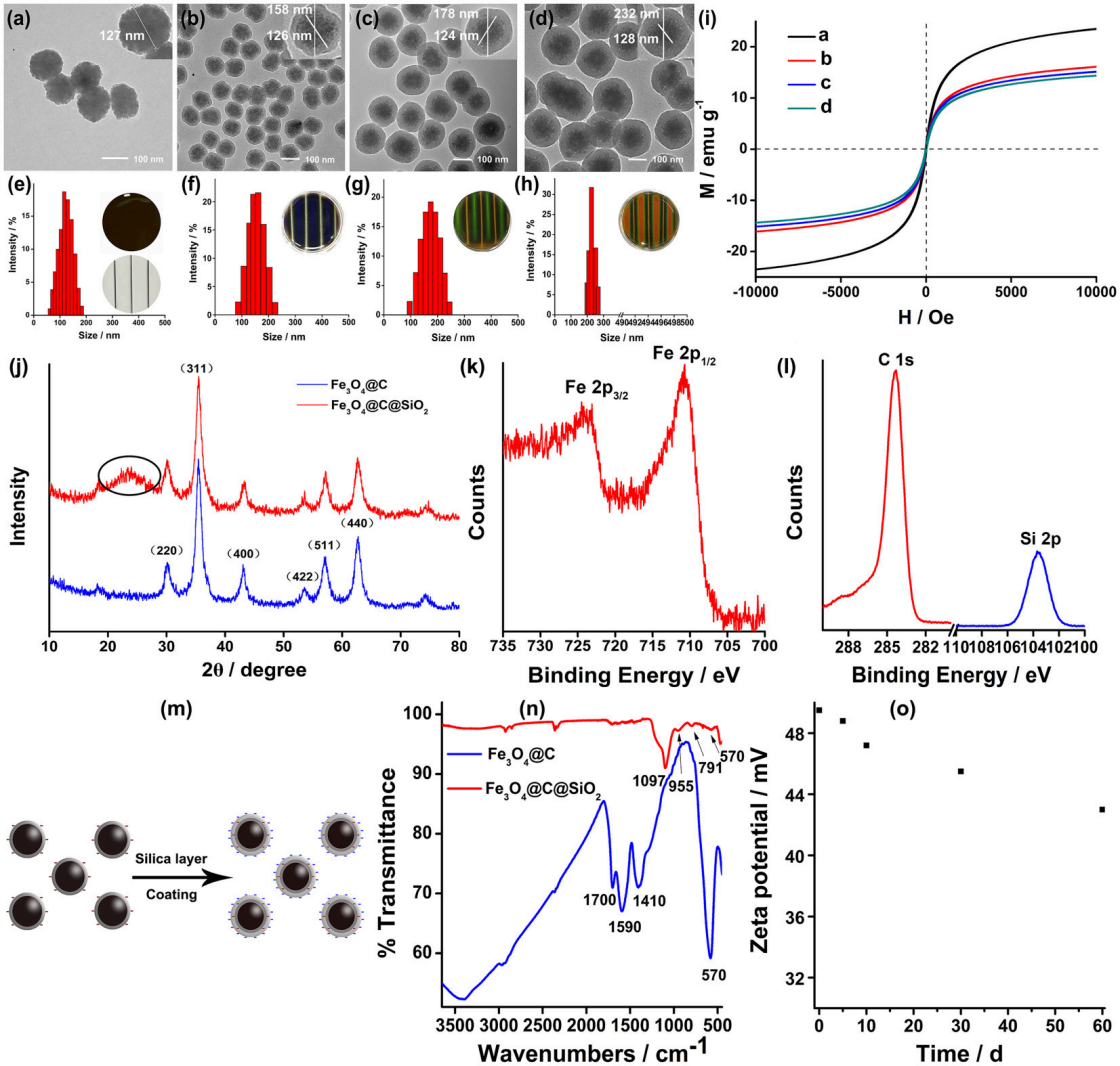
TEM images of Fe_3_O_4_@C **(a)** and Fe_3_O_4_@C@SiO_2_ SMNs **(b–d)**, scale bar: 100 nm. Insets in **(a–d)** were the enlargement of a single synthesized particle, respectively. The size distributions of these SMNs **(e–h)**. The average particle sizes were 127 nm for **(e)**, 158 nm for **(f)**, 175 nm for **(g)** and 230 nm for **(h)**. Insets in **(e–h)**: the corresponding MRPC diffraction color under a laboratory self-made bar-shaped NdFeB magnet array. **(i)** The magnetic hysteresis loop, mass magnetization (M) as a function of the applied EMF (H) measured for these above-mentioned Fe_3_O_4_@C **(a)** and Fe_3_O_4_@C@SiO_2_ **(b–d)** SMNs with different particle sizes at 300 K. **(j)** XRD spectrum of core Fe_3_O_4_@C and Fe_3_O_4_@C@SiO_2_. **(k)** The typical XPS peaks of Fe 2p3/2 and Fe 2p1/2. **(l)** The XPS peaks of C1s (red) and Si2p (blue). **(m)** Scheme of the surface charges of the Fe_3_O_4_@C and Fe_3_O_4_@C@SiO_2_ SMNs; **(n)** FT-IR of Fe_3_O_4_@C and Fe_3_O_4_@C@SiO_2_; **(o)** Zeta potential values of the Fe_3_O_4_@C@SiO_2_ SMNs dispersed in water during storage for 2 months at 4°C.

We employed the sol-gel method for TEOS hydrolysis into SiO_2_ (Ghasemzadeh et al., 2015) by employing TEOS and ammonia as the precursor and catalyst, respectively. The newly formed Fe_3_O_4_@C@SiO_2_ SMNs are composed of a double-shell structure by carbon and the SiO_2_ layer. It is tightly packaged on the surface of the prepared Fe_3_O_4_@C SMNs making it disperse well into the water solution. By adjusting the amount of the catalyst (ammonia) in the reaction system, the thickness of SiO_2_ layer outside the Fe_3_O_4_@C SMNs can be controlled and obtain the appropriate particle size. With the increasing volume of the ammonia from 0.6, 1 to 1.5 mL, the sizes of SMNs were gradually increased. The morphologies and approximate sizes of the Fe_3_O_4_@C and Fe_3_O_4_@C@SiO_2_ SMNs were characterized by transmission electron microscope (TEM) in Figures 1a–d and scanning electron microscope (SEM) in Figure S1. These SMNs were demonstrated uniform with shapes, sizes and the distinct typical core-shell structures. The core/shell ratio was about 2.4-3.4 by calculation and the thickness of the SiO_2_ shell was homogeneous (Figures 1a–d insets).

Under the room temperature (RT) and constant votexing speed, accurate particle size of Fe_3_O_4_@C and Fe_3_O_4_@C@SiO_2_ SMNs could be obtained by DLS, the diameters was 127, 158, 175, and 230 nm (Figures 1e–h), respectively. It also could be observed that different diffraction colors as blue, green and red of the corresponding MRPCs under the same 300 mT EMF. It suggested that, with the growing of the SiO_2_ layer, the size of SMN is correspondingly increased; and the dipole spacing would increase and result in red-shifting of the diffraction color until it turned to colorless (Figures 1e–h insets) under our self-prepared NdFeB strip-type magnet array.

Superparamagnetism of the SMNs is essential for achieving the reversible optical response of PC (Ge and Yin, 2011). As illustrated in Figure 1i, the magnetic hysteresis loops of SMNs were measured at RT. Superparamagnetism and no remanence or coercivity are evaluated by the hysteresis loops between −10,000 and 10,000 Oe. The saturated magnetizations of SMNs-Fe_3_O_4_@C and Fe_3_O_4_@C@SiO_2_ were found to be about 24 and 15 emu g^−1^, respectively. They were slightly decreased with the increasing sizes of the particles because of the lower weight ratio of Fe_3_O_4_ in SMNs. It suggests that our prepared SMNs can be rapidly self-assembled to PC structure under EMF in aqueous solution.

X-Ray diffraction (XRD) confirm the crystalline form of Fe_3_O_4_@C and Fe_3_O_4_@C@SiO_2_ SMNs, which were shown in Figure 1j which were completely coincident with the previous study (Deng et al., 2005) that the reflections peaks of (220) (311) (400) (422) (511) (440) can be indexed to iron oxides (JCPDS no. 75-1609) and the diffraction peaks were narrow. It was also clearly indicated they were consistent with the standard sample of nanocrystalline Fe_3_O_4_ or γ-Fe_2_O_3_, and the crystallinity was satisfied. The primary crystallite size was 11.3 nm calculated from the Scherrer equation (Zhang et al., 2008; Holzwarth and Gibson, 2011; Yang et al., 2016) based on the strongest (311) peak which was smaller than that of the critical size of the superparamagnetic-ferromagnetic transition (30 nm for Fe_3_O_4_). That is the main reason for maintaining the superparamagnetic property of the SMNs to increase their magnetic response and magnetic interaction. It appeared that one peak of Fe_3_O_4_@C@SiO_2_ at 24° (Figure 1j, the black circle in the red line) was different from that of the Fe_3_O_4_@C (Figure 1j, the blue line) in XRD spectrum. We deduced it was the layer of SiO_2_. Since the XRD pattern of Fe_3_O_4_ was similar with γ- Fe_2_O_3_, X-ray photoelectron spectroscopy (XPS) was used to further identify the chemical composition of the Fe_3_O_4_@C@SiO_2_ SMNs. The two photoelectron peaks at binding energy of about 723.3 and 710.5 eV were displayed in Figure 1k which attributed to Fe 2p_3/2_ and Fe 2p_1/2_. There weren't obviously any other peaks in vicinity of both main peaks corresponding to the above-mentioned higher binding energies, and the elements composition ratio was 2:1, which was the obvious characteristic of Fe_3_O_4_. The photoelectron peaks at 284.4 and 103.7eV were corresponding to the typical peaks of C1s and Si2p illustrated in Figure 1k which was consistent with the stoichiometry of carbon and SiO_2_ layer(Figure 1l).

Fe_3_O_4_@C@SiO_2_ SMNs are expected to have appropriate surface properties that are not only with the characteristics of sufficient repulsion to balance the magnetic dipole force during the self-assembling process but also provide the highly hydrophilicity. Figure 1m illustrates the surface electric charges increasing of Fe_3_O_4_@C@SiO_2_ by SiO_2_ layer coating outside the Fe_3_O_4_@C SMNs and formed the double-shell structure composed of carbon and SiO_2_ layer. The surface charges of Fe_3_O_4_@C@SiO_2_ were richer than that of the former one. FT-IR spectroscopy further confirms the synthesis of the obtained products. The main peaks appeared at 570, 1,410, 1,590, 1,700, and 3,200–3,500 cm^−1^ were the distinguished features of the curve of Fe_3_O_4_@C SMNs in FT-IR (Figure 1n, the blue line). The strong bands at around 570 cm^−1^ were different from the absorbing peaks of maghemite at 630 cm^−1^ (Nasrazadani and Raman, 1993), which verified that the core of products was magnetite-Fe_3_O_4_. The stretching vibrations of C-C and C = C were found at 1,410 and 1,590 cm^−1^ that are due to the presence of amorphous carbon (Amendola et al., 2011); at 1,700 cm^−1^, arising from the stretching vibrations of C = O; and the vibration at 3,200–2,500 cm^−1^, could be attributed to the stretching vibrations of -OH group. This wide and scattered absorption peak is an important symbol of carboxylic group. After coating with silica, it could be observed in Figure 1n (the red line) that three peaks at 1,097, 955, and 791 cm^−1^ were corresponding to Si-O-Si, Si-O group and Si-OH groups of Fe_3_O_4_@C@SiO_2_ SMNs. While they did not appear in FT-IR spectrum of that of the Fe_3_O_4_@C SMNs in Figure 1n (the blue line). All the above-mentioned indicated that the surface of the Fe_3_O_4_@C@SiO_2_ SMNs containing a large number of hydrophilic groups reduced the aggregation of the magnetic particles and increased its hydrophilicity. The external SiO_2_ layer could be etched outside the SMNs during storage increasing the permeability of the SiO_2_ layer (Hu et al., 2012). The exposed -COOH of the middle layer compensated the losing of Si-OH in the outer layer. It effectively enhances the charge stability and maintains the long-range electrostatic repulsion. What's more, Si-OH group is more polar and easily modified (Wang and Yin, 2016). Therefore, the water-soluble functionalized SMNs are expected to be wide applying in the fields of bio-separation and bio-detection.

Highly charged SMNs in the size range of 100–200 nm are interesting building blocks for constructing of MRPCs (Yang et al., 2016). One significant challenge for the preparation of MRPCs particles was the consistency of their photonic performances during the storage with aqueous form on account of the charge instability of the prepared Fe_3_O_4_ SMNs (Hu et al., 2012). Compared to the original zeta potential (−50 mV) of the newly prepared SMNs, it was slightly dropped to −43 mV after continuous monitored for a relative long time (6 months) stored at 4°C in water (Figure 1o). The value of zeta potential exceeded the cut-off value (−30 mV) to keep the stability of the SMNs. It is attributed to the formation of Si-O-Si networks and Si-OH groups. Meanwhile, when the silicon layer of the Fe_3_O_4_@C@SiO_2_ SMNs was etched and increased permeability of SiO_2_ layer, the -OH groups were revealed and dissociated from the SMNs surface. However, the exposed -COOH groups of the middle carbon layer compensates for this loss which was consistent with that of the FT-IR and XRD spectrum. The negative charges of Fe_3_O_4_@C@SiO_2_ SMNs can always keep balance during the above processes. Otherwise, we speculate that, the stable carbon layer can decrease the magnetism and prevent the magnetic attraction from the EMF to form the 2D MRPCs.

Table 1 showed the typical methods for the synthesis of MRPCs. Compared with the previous works listed in Table 1, the surface zeta potential is relatively high, the storage time (6 m) is longer than that of our previous work (Tang et al., 2018), and the diffraction wavelength range in water under EMF is moderate as wide as almost in the whole visible spectrum which is appropriate for biomolecular action. The most interesting aspect of the preparation method for the 3D MRPCs is facile, and the prepared Fe_3_O_4_@C@SiO_2_ SMNs can be stably monodispersed in water for 6 m without precipitation and turbidity, and it can be rapidly assembling to MRPCs under EMF within 1 s as fast as that of the PAA (Ge et al., 2007a) and PSSMA-capped (Tang et al., 2018) Fe_3_O_4_ CNC-particle-based and the steric repulsion-based MRPCs (Luo et al., 2014).

**Table 1:** Comparison of the properties of 3D MRPCs.

		**Type of shell**			
	**Nothing**	**Methyl methacrylate**	**SiO_**2**_**	**PSSMA and SiO_**2**_**	**C and SiO_**2**_**
Zeta potential (mV)	−51	−43	−40	−64	−50
Dispersing media	Encapsulated between two glass slides in a liquid (water) film	Polar solvents including water	Ethanol	Water	Water
Diffraction wavelength range under EMF (nm)	230	155	120–150	200	167
Storge time (m)	–	–	–	4	6
References	Ge et al., 2008	Xu et al., 2002	He et al., 2011, 2012a	Tang et al., 2018	This work

“*–” refers to not mentioned yet*.

**Figure 2:**
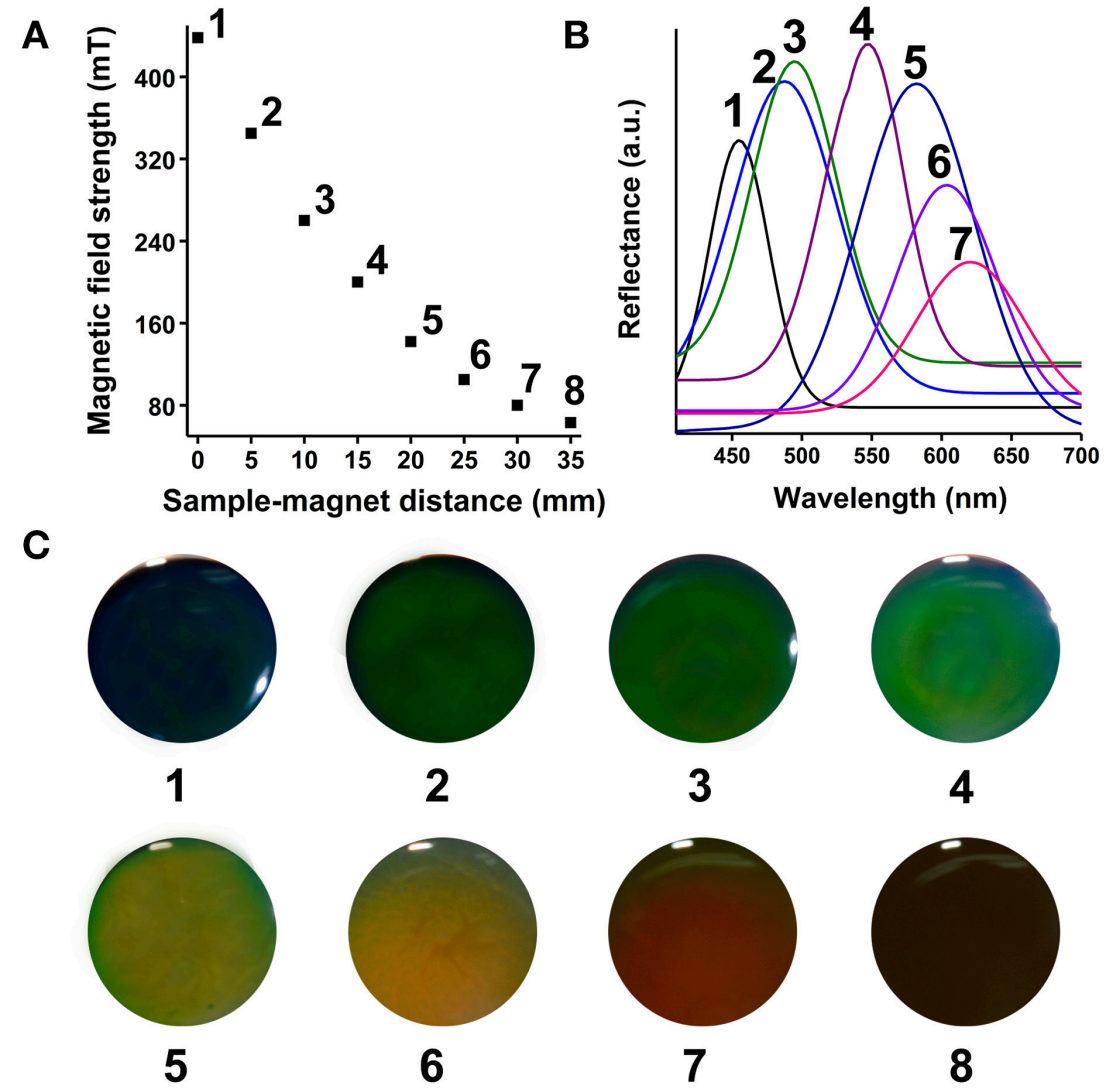
**(A)** Spatial distribution of the sample-magnet distance and magnetic field strength (1–8 different intervals from 0 to 35 mm). **(B)** Reflection spectra of MRPC with magnetic fluid of 175 nm-SMNs on the corresponding EMF of 1–7 intervals. **(C)** Diffraction colors of the formed 3D MRPCs of the 1–8 intervals.

Figure 2A showed the relationship between the sample-magnet distance and EMF intensity (NdFeB magnet). A 1D to 3D phase transition happens within 1s as the concentration of EMF intensity increases. The EMF intensity was monitored by Hall probe and negatively correlated with the distance of the sample-magnet from 0 to 35 mm (1-8 different intervals) by 5 mm gradient-interval. To obtain the satisfied diffraction colors, the SMNs particle sizes were optimized. SMNs with 175 nm were selected and employed to be induced by EMF, because SMNs with too small (158 nm) size or too big size (230 nm) could not acquire full visible spectrum. The MRPC reflection peaks and diffraction colors of the 175 nm-SMNs could be instantly and reversibly induced to 3D MRPCs by tuning upon the change of EMF intensity (Figures 2B,C). The reflection peaks could be effectively tuned between 454 and 621 nm with the intensity of EMF in the range of 438–63 mT. The reflection peaks from left to right were 454, 487, 494, 548, 581, 604, and 621 nm, respectively (Figure 2C, 1–7); diffraction colors were also gradually red-shifted with the decrease of EMF intensity. It could be observed that, when the EMF intensity was smaller than 63 mT, the SMNs were disorderly and no unique PC diffraction color could be visible (Figure 2C, 8). The diffractive color changes of the 3D MRPCs could also be dynamically observed in a beaker (Video S1) by dropping (Video S2) and in a well (Video S3) by tuning the distance between the container and magnet.

**Figure 3:**
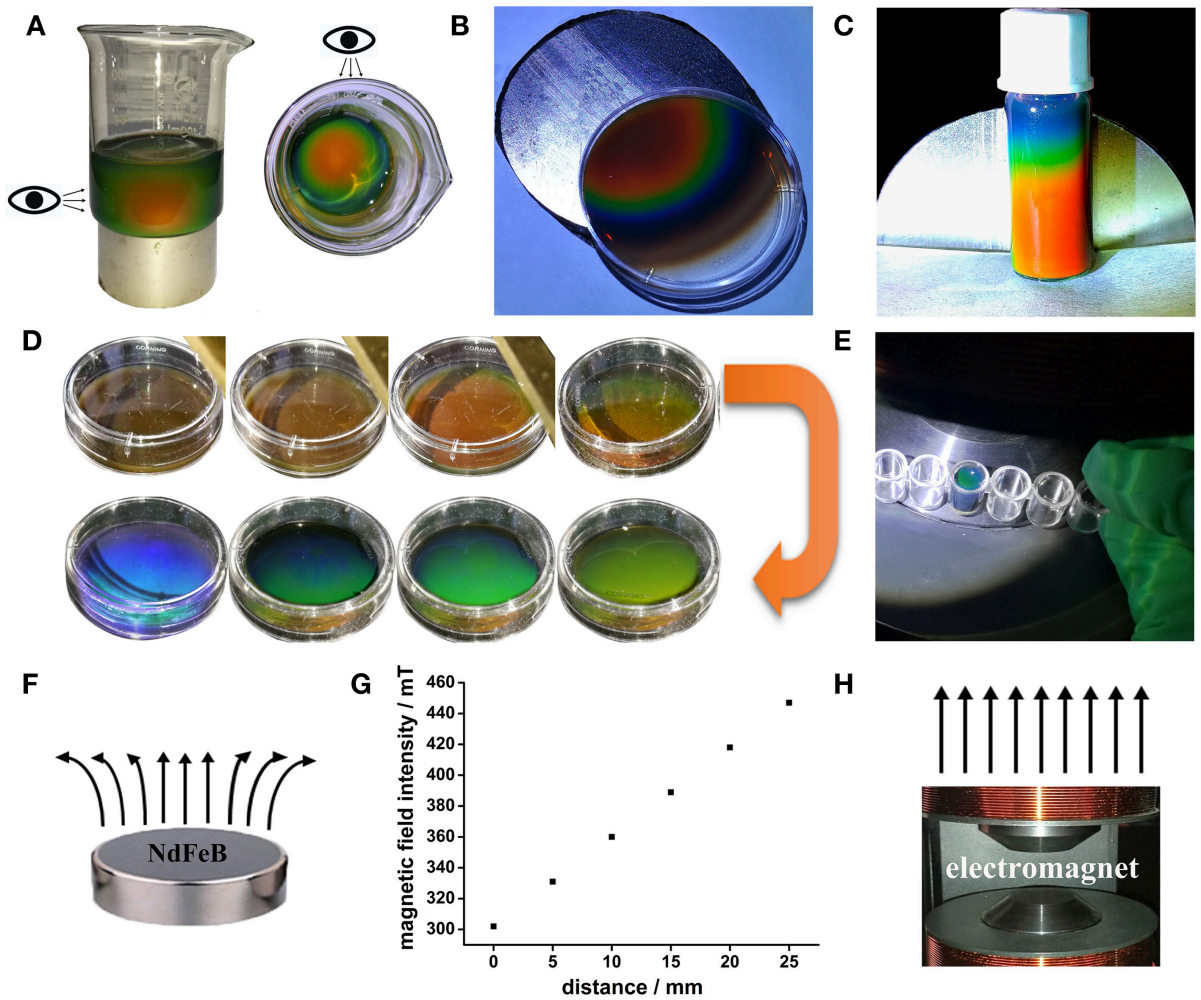
**(A–C)** 3D and rainbow-like MRPCs under cake-like NdFeB magnet. **(D,E)** 3D MRPCs induced by the electromagnet, **(F)** the NdFeB permanent magnet and the corresponding magnetic line, **(G)** relationship between magnetic field strength from center to periphery, **(H)** the electromagnet (polar surface) and the corresponding magnetic line.

The prepared SMNs still could be induced into MRPCs after 2-month storage. Vivid rainbow-like diffraction colors could be successfully induced by the cylindrical NdFeB magnet (Figures 3A–C) of the prepared SMNs and formed into 3D MRPCs. The side and the top view proved that the color of the structure is independent with the angle of view, which presenting its excellent 3D structure (Figure 3A). Intensities of EMF could be adjusted by changing the current of the electromagnet generator (Figure S2). It could be observed that changes of the different structure diffraction colors and uniform diffraction color of the 3D MRPCs (Figures 3D,E) under it with the increasing of current (Video S4). The result as the same as that under inducing of NdFeB magnet. It revealed that the SMNs started to be self-assembling, and the diffractive color appeared, then the color came to blue-shift under inducing of the EMF from 0 to 450 mT with the enhancing of the current. The 3D MRPCs have good geometric symmetry and photonic band gaps which were completely overlapping. The MF lines of NdFeB magnet is gradually diverging from the center to the periphery (Figure 3F); and the EMF strength of the NdFeB is gradually enhanced from middle to edge of the magnet (Figure 3G). With the influence of the gradually and gradiently increasing of EMF to the magnetic fluids, the magnetic dipole space became narrow from the center to the surrounding, the diffraction color of the 3D MRPCs was gradually blue-shifted. Therefore, it resulted in rainbow-like MRPCs of the MF with a gradient of magnetic dipole spacing in the same container. Similarly, it presented in a single and uniform diffraction color when the magnetic dipole space was the same, i.e., the magnetic fluid were under our self-made electromagnetic field which could be considered as a uniform EMF (Figure 3H).

**Figure 4:**
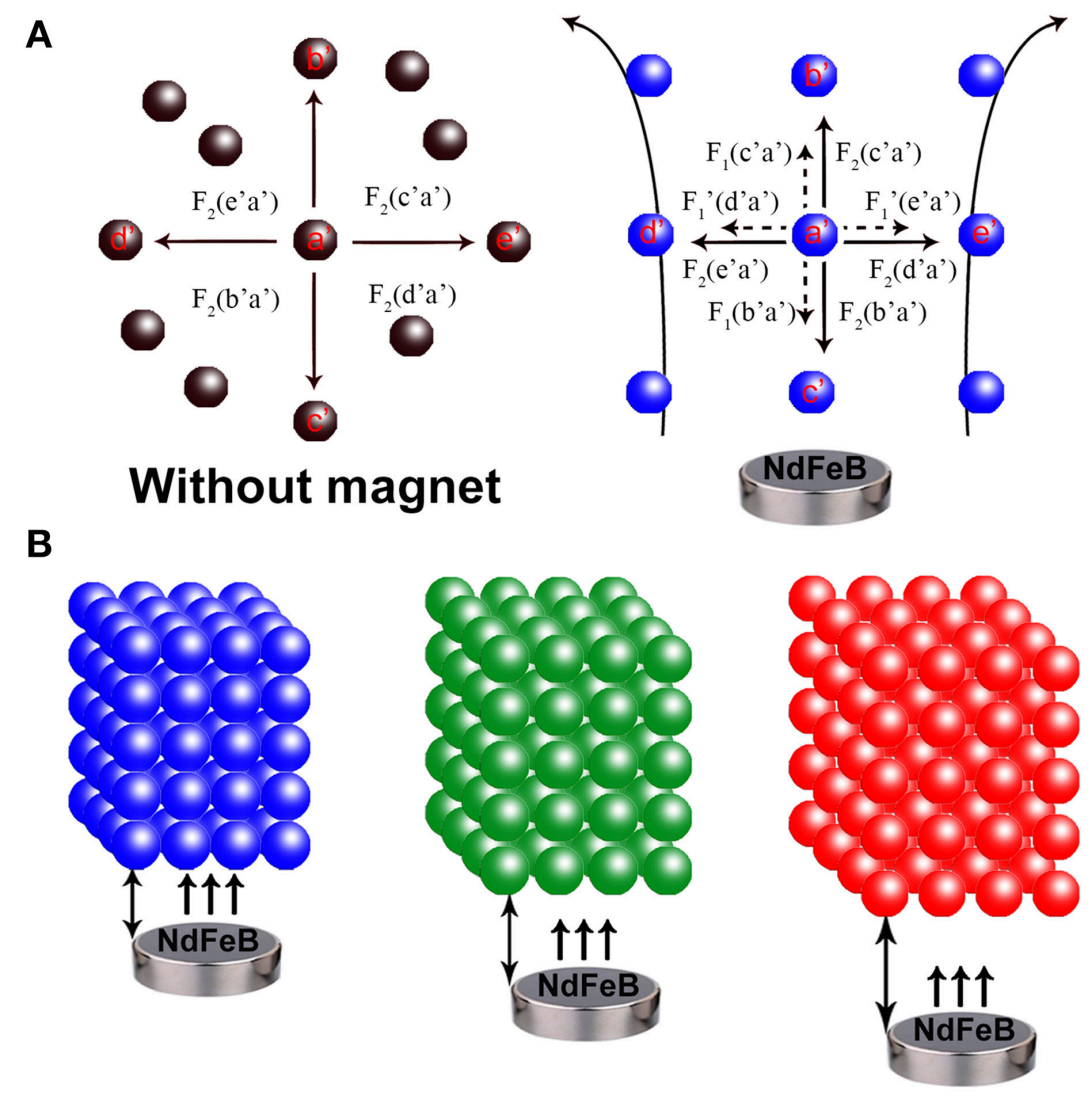
**(A)** Schematic illustration of the forces on Fe_3_O_4_@C@SiO_2_ SMNs in the absence (left) and presence of an EMF (right). F_1_: dipole-dipole attractive force (the small dotted arrow), ${{\text{F}}_{1}}^{\prime }$: dipole-dipole repulsive force (the small dotted arrow), F_2_: electrostatic repulsive force (the solid arrow). a′ to e′ refers to the different directions. **(B)** Assembling a 3D orderly array under the EMF. The MRPCs diffraction was correspondingly red-shift from left to right as blue, green and red with the SMNs-magnet distance gradually increased.

The assembling behavior and mechanism of the 3D MRPCs under EMF can be monitored employing the dark optical microscope with laboratory modification (Figure S3). The assembly SMNs can be formed to 1D ordered chain-like structures from random state and then further evolve into multi-chains and the long-range 2D planar/sheet structures. Further increasing of the SMNs concentration and the interparticle potential may lead to coalescence of the 2D planar structures and eventually assemble to 3D crystals (Ge et al., 2011; Wang et al., 2013; Wang and Yin, 2016). A thin film of magnetic fluid between two glass slides was placed on the microscope. A movable magnet was vertically placed on stage underneath the sample slides, and the EMF could be conveniently controlled by tuning the distance between the glasses and magnet. Originally, in the absence of EMF, these SMNs were randomly dispersed and scattered distribution (Brown movement) in aqueous solution by the strong electrostatic repulsive interactions and the color in the visual field turned to almost brown (left panel of Figure 4A). The electrostatic repulsion force of the SMNs can be expressed as F_2_. The SMNs are magnetized and produce the dipole moment when EMF is applied. The force between the dipoles can be expressed as F_1_. θ is the angle between magnetic field lines and dipole connection. When the angle between dipole-dipole connection and magnetic field lines is 0° ≤ θ < 54.09°, the dipole attractive force is *F*_1_ = −6(*m*^2^/*d*^4^), which causes the dipoles to attract with each other and assemble into chains. When 54.09° < θ ≤ 90°, the dipole repulsive force is ${F_{1}}^{\prime }$ = 3(*m*^2^/*d*^4^), constitute 1D chain-like structures are mutually exclusive to minimize their energy which can be easily manipulated by EMF. The key point for the assembling of MRPCs in aqueous solution is to establish a balance among the dipole-dipole attractive force, exclusion force and dipole-dipole electrostatic repulsive forces (Wang and Yin, 2016).

**Figure 5:**
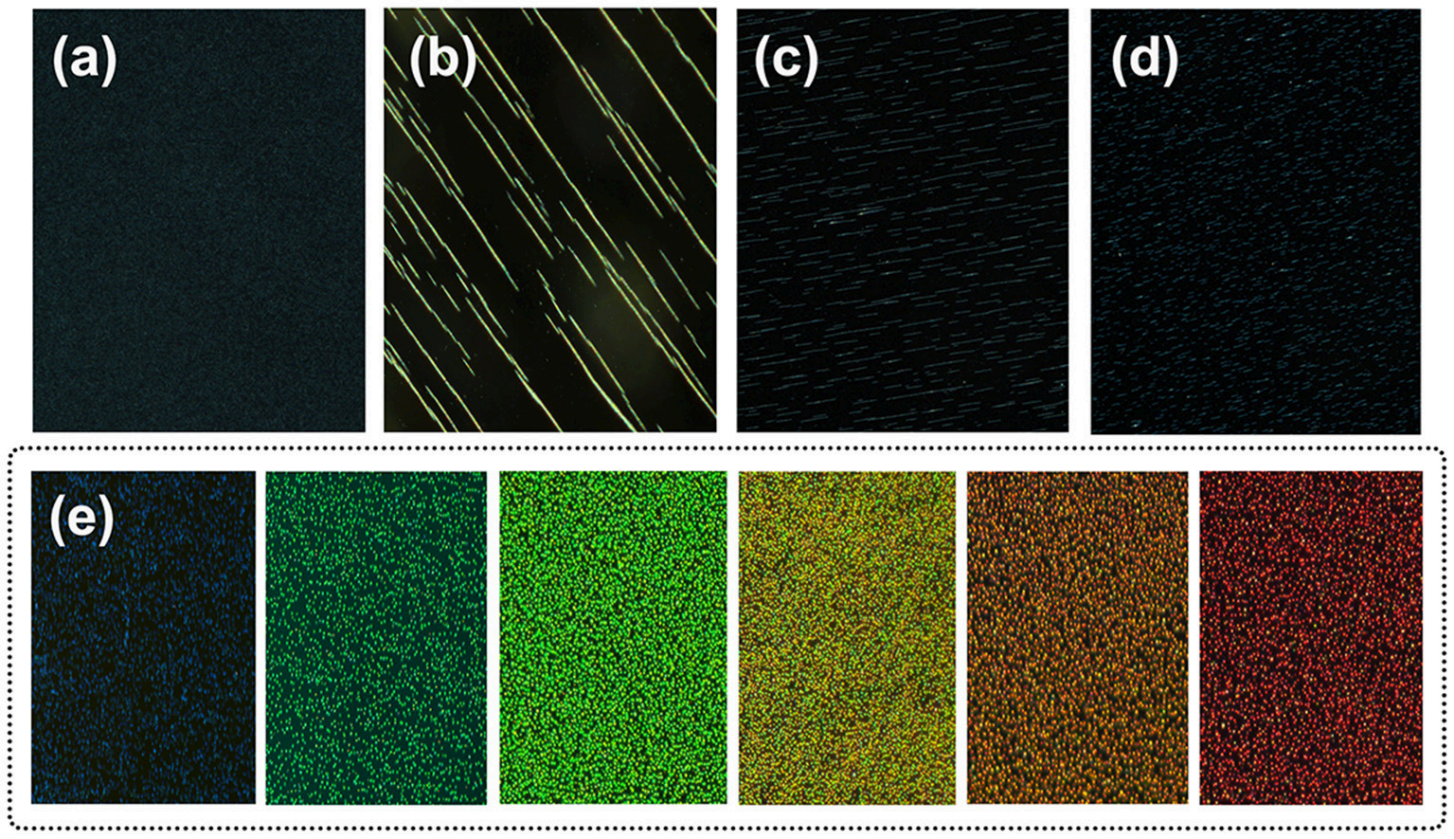
Dark-field optical microscopy images of MRPC. **(a)** Image of the magnetic fluid without EMF. **(b–d)** Image of the SMNs self-assemble into chains. **(e)** The different reflection colors of MRPC.

The macroscopic manifestation by the naked eyes revealed that the SMNs with a low concentration could be assembled into chains by applying the tilted EMF when the dipole-dipole attractive force overcomes the electrostatic repulsive forces along the direction of the magnetic field lines (Figure 5b) compared with that without EMF (Figure 5a). Figures 5c,d showed that when the concentration of the magnetic fluid increases, the chains became shorter and close to each other. When strong EMF was applied to a relatively high concentration of SMNs (Geng et al., 2015), the distances between the chains and the particles distance in chain are consistent, and the orderly 3D MRPC array structure was to be assembled (Whitesides and Boncheva, 2002), with vivid diffraction colors (Figures 3A,B). According to the Bragg diffraction equation:

$$\begin{array}{r} \text{m}\lambda =2\text{nd}\sin\theta \end{array}$$

The different diffraction wavelength of MRPC could be obtained by changing the distance of microspheres (*d*), which could be adjusted by tuning the particle size or EMF intensity, (Yang et al., 2016) such as gradually weakening the EMF by increasing the distance between the glasses and magnet in our work. The color from dark blue to green, lime, olive, orange, and red followed along with the increase of the magnet-sample distance from left to right (Figure 5e). The photonic bandgaps of our prepared 3D MRPCs at all angles are completely overlapped characterized with a wider complete photonic band-gap and high optical quality (Wong et al., 2003; Cai et al., 2012). It can be considered as the true sense of 3D MRPCs and the diffraction color is not completely influenced by the observer's view of angle which means the diffractive color remains the same at any angle.

## Conclusions

In summary, we have prepared novel, extremely stable and biocompatible 3D MRPCs under different kinds of EMFs; and the sizes of the SMNs are easily controllable by adjusting the amount of catalyst. They can rapidly respond under EMF with fast and reversibly tunable diffractions in the visible range with full visible and various colors and also be obtained by changing the intensity of EMF. The diffractive color changes of the 3D MRPCs can also be dynamically observed in various containers under both permanent magnets and electromagnets. They can be employed as a super energy-efficient and infinitely recyclable omni-directional display device, optical sensors, optical switches and identification of authenticity. The desired indicator or display content could be easily adjusted when the shape or strength of EMF is changed. Due to the advantage of the long-term stability in aqueous solution, it is not only conducive to optical display but also to the biological applications. It can be assumed that our prepared hydrophilic SMNs are promising to be employed for accurate release of drug to target (*in vivo*) and the reaction/treatment is carried out or started by using the optical properties of the PCs.

## Author Contributions

HL, PW, and NL: experimental design, data analysis and interpretation, manuscript writing, and manuscript revision; CW and QD: material synthesis and characterizations, data acquisition.

### Conflict of Interest Statement

The authors declare that the research was conducted in the absence of any commercial or financial relationships that could be construed as a potential conflict of interest.
