# Supplementary material for: A Two-Step Strategy for Fabrication of Biocompatible 3D Magnetically Responsive Photonic Crystals
Source: Front Chem. 2019 Feb 1;7:26. doi: 10.3389/fchem.2019.00026 (PMC6367226; doi:10.3389/fchem.2019.00026)
Supplement: Supplementary file 5 [file Data_Sheet_1.PDF]

Supporting Information

A Two-step Strategy for Fabrication of Biocompatible 3D Magnetically Responsive Photonic Crystals

Hui Liu[1], Caiqin Wang[1], Peixi Wang[2], Nan Liu[1, 2*] and Qingfeng Du [2*]

1. School of Public Health, Lanzhou University, Lanzhou, 730000, P. R. China

2. General Practice Center, Nanhai Hospital, Southern Medical University, Foshan, 528244, P. R. China

* Corresponding author (email: 13688869875@163.com and nhyyqk@126.com)

Supporting Figures

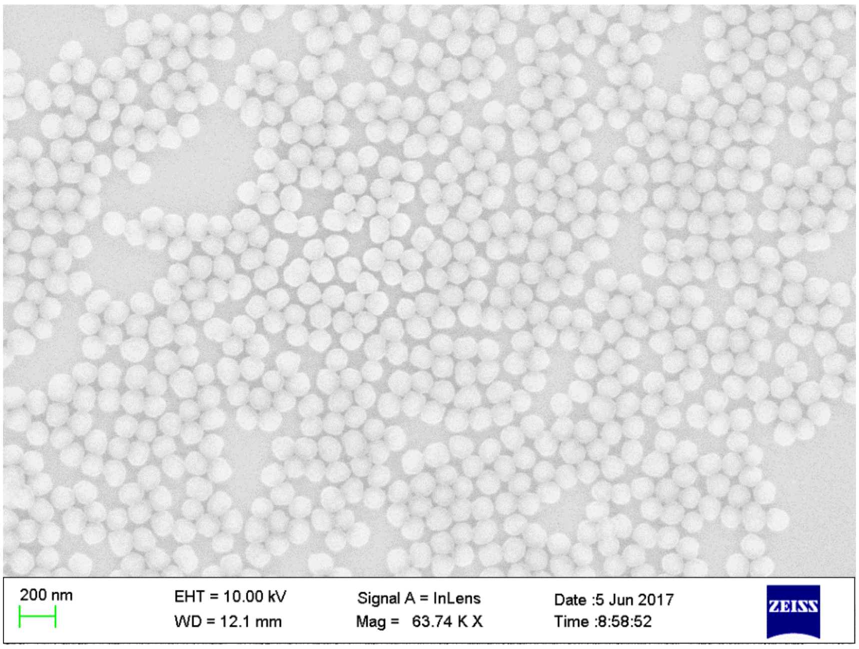

Figure S1. SEM images of Fe_3O_4@C@SiO_2 SMNs

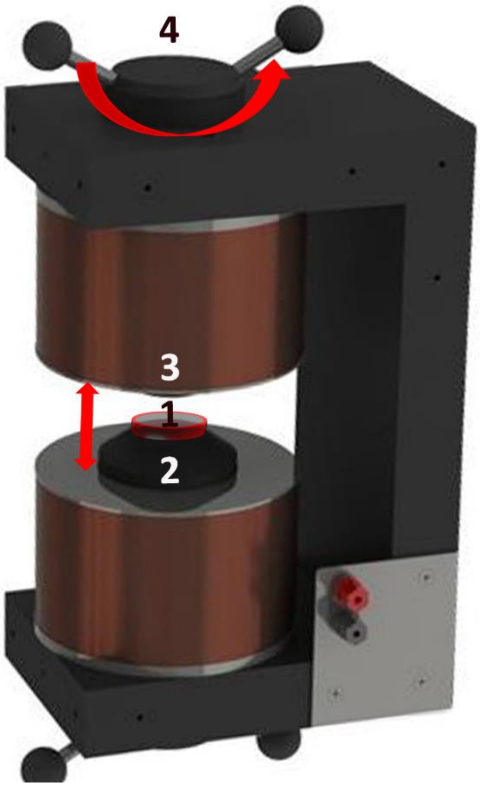

Figure S2. The laboratory-made electromagnet

The movable laboratory-made electromagnetic field generator is formed by two electromagnets (made of Helmholtz loops). The 3 D MRPCs in the watchglass (1) are placed on a fixed laboratory-made electromagnet polar surface (2). The other electromagnet (3) is vertically placed on the watchglass. The electromagnetic field generator could be conveniently controlled by tuning the distance, i.e. the handle (4) between the MRPCs and the two electromagnets. The EMF intensity of electromagnetic field can be tuned by adjust electric current. The different structure diffraction color changes of the 3 D MRPCs can be observed. The effects induced by the laboratory-made electromagnetic field generator are as same as that of NdFeB. Along with the current increasing, the SMNs start to be assembling and the diffraction color is rapidly blue-shift. Simultaneously, the electromagnetic field possess the function of automatically reversing the magnetic field direction which means that the EMF can be gradually and periodically generated from -4500 Gs to 0 and then increased to +4500 Gs. The diffraction color gradually red shift (from blue to red), eventually to colorless, and then gradually from colorless to blue. The dynamic diffraction color changes can be observed in Video S 4.

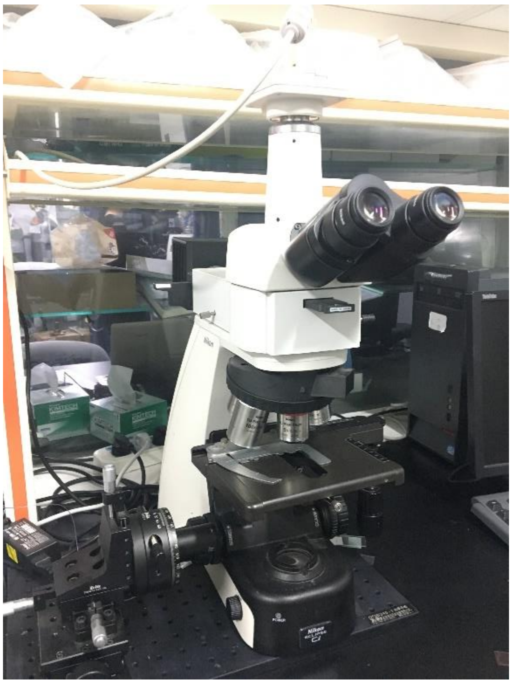

Figure S3. The dark-field optical microscopy by some laboratory modification for the observation of the 3D MRPCs assembling process.

Supporting Videos

Video S1 The color changes of the 3D MRPCs.

The Fe_3O_4@C@SiO_2 SMNs solution was placed in a beaker. A movable NdFeB cylinder-shaped magnet was vertically placed underneath the beaker, and the magnet could be conveniently controlled by tuning the distance between the beaker and magnet.

Video S 2 The color changes of the 3D MRPCs.

The Fe_3O_4@C@SiO_2 SMNs solution was dropped on the glass sheet and the color changed to blue immediately to form the 3D MRPCs. And then, the glass sheet was raised from the magnet underneath. The color turned to gray again.

Video S3 The color changes of the 3D MRPCs.

The Fe_3O_4@C@SiO_2 SMNs solution was placed in a well of the 96-well plate. A movable NdFeB cylinder-shaped magnet was vertically placed underneath the well, and the magnet could be conveniently controlled by tuning the distance between the well and magnet.

Video S4 The color changes of the 3D MRPCs.

The Fe_3O_4@C@SiO_2 SMNs solution was placed in a well of the 96-well plate. And the well was close to the laboratory-made electromagnetic field generator. The color changed immediately along with the change of electromagnet intensity.
